# Therapeutic Efficacy of Ultrasound-Guided Selective Nerve Block on Chronic Cervical Radiculopathy

**DOI:** 10.3390/medicina60061002

**Published:** 2024-06-19

**Authors:** Hyo Jin Joo, Seongmin Choi, Byoung Hoon Kim, Min-Su Kim, Ga Yang Shim, Sung Joon Chung, Jinmann Chon, Myung Chul Yoo, Yunsoo Soh

**Affiliations:** 1 Department of Physical Medicine & Rehabilitation, College of Medicine, Kyung Hee University, Seoul 02447, Republic of Korea; hjjoo94@gmail.com (H.J.J.); ysisminee@naver.com (S.C.); 26877@khmc.or.kr (G.Y.S.); kkangmann@naver.com (J.C.); 2 Department of Physical Medicine & Rehabilitation, Kyung Hee University Medical Center, Seoul 02447, Republic of Korea

**Keywords:** cervical radiculopathy, spinal nerve roots, selective nerve root block, ultrasonography

## Abstract

*Background and Objectives*: Cervical radiculopathy (CR) manifests as pain and sensorimotor disturbances in the upper extremities, often resulting from nerve root compression due to intervertebral disc herniation, degenerative changes, or trauma. While conservative treatments are initially preferred, persistent or severe cases may require surgical intervention. Ultrasound-guided selective nerve root block (SNRB) has emerged as a promising intervention for alleviating symptoms and potentially obviating the need for surgery. This study evaluates the therapeutic efficacy of ultrasound-guided SNRB in managing chronic CR, aiming to determine its potential in symptom relief and delaying or avoiding surgical procedures. *Materials and Methods*: A retrospective analysis was conducted on 720 outpatients treated for CR between October 2019 and March 2022. After excluding patients with traumatic CR, previous surgeries, malignancies, progressive neurological symptoms requiring immediate surgery, or inadequate conservative treatment, 92 patients who had experienced cervical radicular pain for more than three months and had failed to improve after more than six weeks of conservative treatment with VAS scores ≥ 5 were included. The patients underwent single or multiple ultrasound-guided SNRB procedures, involving the injection of dexamethasone and lidocaine under real-time ultrasound guidance. Symptom severity was assessed at the baseline, and at 4, 8, and 12 weeks post-procedure using the Visual Analog Scale (VAS). The data collected included age, sex, presence of neck and/or radicular pain, physical examination findings, recurrence of symptoms, improvement in symptoms, and whether surgical intervention was ultimately required. Statistical analyses were performed to identify the factors associated with symptom improvement or recurrence. *Results*: Significant symptom improvement was observed in 69 (75.0%) participants post-SNRB, with 55 (79.7%) showing improvement at 4 weeks, 11 (15.9%) at 8 weeks, and 3 (4.4%) at 12 weeks. Symptom recurrence, defined by an increase in VAS score accompanied by a pain flare lasting at least 24 h after a pain-free interval of at least one month, was noted in 48 (52.2%) patients. The presence of combined neck and radicular pain was a significant predictor of recurrence (*p* = 0.008). No significant associations were found between symptom relief and factors such as age, gender, initial pain severity, or MRI findings. *Conclusions*: Ultrasound-guided SNRB effectively manages chronic CR, providing substantial symptom relief and potentially reducing the need for surgical intervention. This technique offers a promising conservative treatment option, especially given its real-time visualization advantages and minimal radiation exposure.

## 1. Introduction

Cervical radiculopathy (CR) is characterized by pain and sensorimotor symptoms in the upper extremities, affecting one or both sides of a specific cervical nerve root [1]. It presents as radicular pain in the neck and arm, accompanied by numbness, tingling, and weakness, primarily in the affected limb. These symptoms typically result from the compression or irritation of the nerve root, which can stem from various conditions such as disc herniation, ligament hypertrophy, facet joint enlargement, tumors, or trauma [2]. Approximately 70–75% of CR cases co-occur with degenerative changes, while 20–25% are attributed to intervertebral disc herniations [3]. Repetitive overloads and micro-injuries lead to spinal canal narrowing and disc degeneration via the hypertrophy of the spinal joints and the encroachment of surrounding structures. This degeneration promotes osteophyte formation, elevated levels of local prostaglandins causing inflammation near the dorsal root ganglion (DRG) [4]. Additionally, factors such as trauma, infection, and tumors can reduce the available space for the exiting nerve root, resulting in radicular symptoms [5].

In the management of CR, initial treatment strategies typically favor a conservative approach. Nonsurgical options include immobilization, the administration of nonsteroidal anti-inflammatory drugs (NSAIDs), muscle relaxants, cervical traction, physiotherapy, and epidural steroid injections [6]. It has been reported that conservative management leads to symptom improvement in up to 43% of patients [7,8]. Additionally, Cyteval et al. found that CT-guided nerve root block provided pain relief in 60% of patients who did not respond to more than one month of medical therapy [9]. Surgical intervention is reserved for patients experiencing persistent radicular pain, significant muscle weakness, or signs of myelopathy despite undergoing conservative treatment for over 6 weeks [10]. Surgical options encompass anterior cervical decompression and fusion (ACDF), cervical disc arthroplasty, and posterior foraminotomy. However, surgical procedures carry inherent risks such as spinal cord injury, nerve root damage, transient dysphagia, vertebral artery injury, and postoperative infection [11]. Consequently, in cases lacking urgent ‘red flag’ symptoms, the exploration of alternative nonsurgical options should be considered.

Selective nerve root block (SNRB) serves both diagnostic and therapeutic purposes in managing CR and can be precisely administered under imaging guidance such as fluoroscopy or ultrasound [12]. This procedure involves injecting a combination of a local anesthetic and a corticosteroid, targeting the multifaceted pathophysiological mechanisms underlying CR. Corticosteroids administered near the dorsal root ganglion (DRG) and the affected nerve root have shown therapeutic efficacy by directly addressing inflammatory and pain-inducing pathways [13,14]. They achieve this by inhibiting the synthesis of arachidonic acid, a precursor of proinflammatory mediators, thereby interrupting the cascade leading to pain production. When injected into the epidural space, corticosteroids reduce inflammation and mechanically displace the dura mater. This displacement may alleviate neural adhesions and pain. The dual mechanical and pharmacological mode of action underscores the multifaceted role of corticosteroids in managing the radicular pain associated with CR.

Since Galiano et al. first reported the efficacy of ultrasound (US)-guided SNRB in 2005, this technique has gained widespread acceptance and is now considered a viable alternative to the traditional fluoroscopy-guided approach [15]. Ultrasonographic guidance for SNRB offers several distinct advantages over fluoroscopic guidance, particularly in the lower cervical spine. These include eliminating radiation exposure, reducing the risk of arterial needle injury, and enabling real-time dynamic visualization of anatomical structures. These benefits underscore the increasing preference for the ultrasound-guided administration of SNRB [3].

Both surgical intervention and conservative treatment are effective strategies for managing CR. However, Persson et al. and others have reported no significant differences in outcomes between surgical and conservative management of cervical radiculopathy, and conflicting results exist [16,17]. To date, there are few reports directly comparing the outcomes of surgical discectomy and conservative managements, particularly those involving ultrasound-guided interventions. Accordingly, this study aimed to evaluate the therapeutic effectiveness of US-guided cervical SNRB as a means of providing sustained relief and potentially delaying or obviating the need for surgery in patients with chronic CR.

## 2. Materials and Methods

### 2.1. Study Population

**Figure 1:**
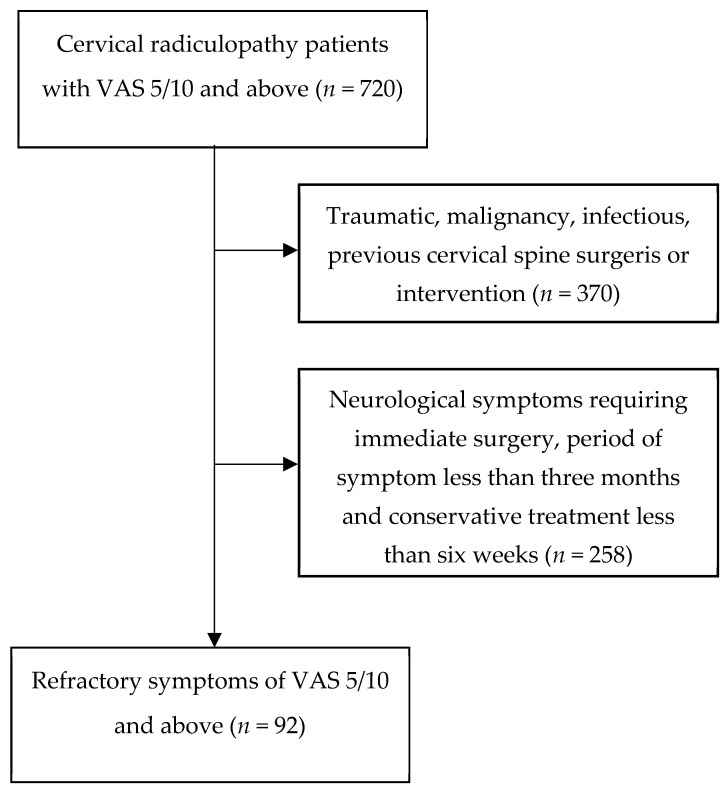
Flowdiagram of this study. A total of 720 patients with pain scores of 5 or more on the VAS for three months or more were treated at the clinic from October 2019 to March 2022. Patients with a history of trauma, cervical surgery, cancer, inflammatory diseases, or progressive neurological symptoms, low adherence were excluded from the study (*n* = 258), resulting in a final sample of 92 patients.

This retrospective study included 720 outpatients who underwent treatment for cervical radiculopathy (CR) between October 2019 and March 2022. CR was defined by the presence of posterior neck pain and/or radicular upper extremity pain, along with sensory and/or motor symptoms attributed to cervical disc herniation or nerve compression at the same nerve root level, as confirmed by cervical MRI with a resolution of 3.0 T. Initially, patients aged 20 years or older with symptoms persisting for more than 3 months and with a Visual Analog Scale (VAS) score of 5 or higher were recruited (*n* = 720). The exclusion criteria included traumatic CR, a history of cervical spine surgeries or interventions, malignancies, inflammatory diseases, and neurological symptoms such as progressive motor weakness requiring immediate surgery. Conservative treatment strategies involved education on proper neck posture and the administration of oral medications, such as NSAIDs and muscle relaxants, applied consistently for at least six weeks. Patients who did not receive this treatment were excluded (*n* = 258). Subsequently, patients who experienced persistent cervical neck and/or radicular pain for more than three months and failed to improve after six weeks of conservative treatment were included if their VAS score remained five or above. Ultimately, 92 patients with intractable chronic CR unresponsive to conservative treatment were recruited for the study (Figure 1).

### 2.2. Study Design and Methodology

Age, sex, severity of pain at four time points (initial, four, eight, and 12 weeks after the procedure), presentation of post neck and/or radicular pain, physical examination, recurrence, improvement, and whether surgery was performed were evaluated. To assess the therapeutic effect, the Faces Pain Scale was used to measure the intensity of pain experienced by patients, expressed on a 0–10 Visual Analog Scale (VAS). Based on the studies by A.M. Boonstra et al. [18] and M.P. Jensen et al. [19] on the classification of VAS scores, a VAS score of 1–4 was classified as mild pain, 5–6 as moderate pain, and 7 or higher as severe pain. During the study period, a follow-up VAS score decrease of 50% or more compared to the initial VAS score was considered an improvement in symptoms. A VAS score of 3/10 or less and a significant symptom reduction of less than 50% of the initial VAS score were also considered to indicate symptom improvement. The recurrence of symptoms was defined as an increase in the VAS score compared to the previous follow-up, accompanied by pain flares lasting at least 24 h, followed by a pain-free condition for at least one month.

### 2.3. Intervention

US-guided cervical SNRB was performed on outpatients by trained physicians. Patients were positioned in the lateral decubitus position on the affected side, with the chin slightly lifted approximately 0–30°. Ultrasound imaging (Samsung Medison, Seoul, Republic of Korea, 612 MHz linear probe) was used to identify the cervical spine level based on the shape of the seventh cervical transverse process, which exhibited a rudimentary anterior tubercle and a prominent posterior tubercle. By moving the probe upward, the target nerve root was located, considering that C5 and C6 possess both anterior and posterior tubercles. The procedure was performed using an ultrasonographic model HS50 (Samsung Medison, Seoul, Republic of Korea) with a 612 MHz linear probe. Patients were positioned in the lateral decubitus position with the affected side up, and the transducer was placed transversely to the lateral aspect of the neck. The cervical spine level was determined by identifying the seventh cervical transverse process, characterized by a rudimentary anterior tubercle and a prominent posterior tubercle. Using power and color Doppler modes, images of the target nerve root, location of the radicular artery, and surrounding vessels were obtained. Following the confirmation of a safe needle pathway, a 50 mm 26 G needle was inserted toward the nerve root using an in-plane approach from posterior to anterior direction. The needle tip was positioned on the dorsal side of the nerve between the posterior tubercle and the target nerve root, with caution taken to avoid damage to the deep vertebral and/or carotid arteries near the injection site. Under real-time ultrasound guidance, the needle tip position was confirmed, and aspiration was performed to avoid blood vessels. Subsequently, 2 cc of a therapeutic mixture consisting of 5 mg dexamethasone and 0.5% lidocaine was carefully injected. All patients received one or multiple (up to 3) consecutive cervical SNRB injections with a 2-week interval between injections. Subsequent injections were performed based on patient symptoms and conditions considering factors such as patient-reported worsening pain or weakness, refusal to undergo surgery, and the decision not to administer a second injection.

### 2.4. Statistical Analysis

Factors associated with patient improvement or recurrence were analyzed using Pearson’s chi-squared test. Univariate logistic regression analyses were conducted to identify predictors of outcomes, presenting odds ratios (ORs) with corresponding 95% confidence intervals (CIs). Data analysis was performed using SPSS (version 23.0; IBM Corp., Armonk, NY, USA). Statistical significance was set at *p* < 0.05.

## 3. Results

### 3.1. Baseline Characteristics

The baseline characteristics of the study population are depicted in Table 1. Among the 92 patients assessed, 69 (75.0%) exhibited symptom improvement, defined as a 50% or greater decrease in the Visual Analog Scale (VAS) score following the procedure. Conversely, 16 patients (17.3%) underwent surgery during the follow-up period, while the remaining 7 patients (7.6%) did not show any improvement but chose not to undergo surgical intervention. The age distribution was predominantly within the 50s and 60s age groups (*n* = 25, 27.2% and *n* = 26, 28.3%, respectively), with a gender distribution of 48 females (52.2%) and 44 males (47.8%). Cervical MRI findings revealed that 39 (42.4%) patients presented with disc herniation only, 44 (47.8%) with both disc herniation and foraminal stenosis, and 9 (9.8%) with disc herniation accompanied by myelopathy. Among these patients, 22 (23.9%) reported a severe pain VAS score of 7 or more.

### 3.2. Results of Statistical Analysis

A total of 69 participants experienced symptom improvement following the initial injection. This represents 75% of the total sample (*n* = 69). Follow-up assessments conducted at 4, 8, and 12 weeks revealed that 55 participants (79.7%) continued to show symptom improvement at 4 weeks, 11 participants (15.9%) at 8 weeks, and 3 participants (4.4%) at 12 weeks. Recurrence was observed in 48 participants (52.2%) who experienced a pain-free period of at least one month followed by an increase in VAS score or pain lasting for 24 h or more. Analysis revealed that the coexistence of neck and radicular pain was a highly significant predictor of symptom recurrence (95% CI, *p* = 0.008). Conversely, symptom relief was not associated with the site of patient complaints or cervical MRI findings (Table 2).

Table 3 presents the results of the univariate logistic regression analysis investigating the impact of various initial patient factors on the likelihood of symptom improvement or recurrence. No statistical significance was found between patient factors, including age, sex, initial pain severity (measured by VAS), presence of motor weakness, sensory symptoms (hypoesthesia), Spurling sign, and shoulder abduction relief sign, and patient outcomes.

## 4. Discussion

Despite initial conservative management like medication and physical therapy, surgery might be considered if patients experience uncontrolled cervical radicular pain, progressive motor weakness, myelopathy symptoms, or persistent spinal instability despite more than 6 weeks of conservative treatment [20]. However, due to the less favorable risk-benefit profile of surgery, numerous studies and guidelines advocate for interventional approaches, particularly epidural steroid injections [21,22]. Fluoroscopy-guided transforaminal epidural steroid injection (TFSEI) is a common procedure, but ultrasound-guided selective nerve root block (SNRB) has emerged as a viable alternative.

Several studies have compared ultrasound and fluoroscopy-guided SNRB [12]. Narouze et al. reported reduced vessel damage with US-guided SNRB by precisely positioning the needle tip near the nerve root [23]. Another recent study comparing clinical outcomes of 120 patients with both methods found no significant difference in effectiveness. However, five cases of intravascular injection occurred in the fluoroscopy group, a potentially serious complication like vertebral dissection or brain/spinal cord infarction [24,25,26]. Wakeling et al. suggested reduced radiation exposure with ultrasound-guided SNRB [27]. These findings indicate similar treatment effects between the two methods. While fluoroscopy-guided cervical epidural steroid injection has been the mainstay treatment, US-guided SNRB is gaining traction as a conservative option due to its real-time visualization and minimized radiation exposure [28].

The findings of our study further strengthen the evidence for US-guided cervical SNRB as a less invasive intervention and an effective nonsurgical treatment, providing valuable insights. In this retrospective study, 75% of eligible surgical candidates could avoid or delay surgery for at least 6 months with US-guided SNRB treatment. Our results demonstrated significant symptom improvement in most patients following post-cervical SNRB, with a notable decrease or delay in surgical intervention. This is particularly relevant considering the ongoing debate about the comparative effectiveness of conservative and surgical approaches for cervical radiculopathy (CR). Kolstad et al. conducted a cohort study on patients with relative indications for surgery, similar to those in our study, and reported that 5 out of 21 patients (24%) experienced symptom relief, including recovery of sensation and muscle strength, leading to the cancellation of surgery after undergoing selective nerve root block [29,30]. Our study demonstrated similar results, showing that US-guided selective nerve root block was performed on patients who did not respond to more than 6 weeks of conservative treatment and were considered for surgery. This distinguishes our study from previous research.

In addition, this study analyzed the relationship between various factors and the effectiveness of US-guided selective nerve root block to determine which patient groups benefit most from this procedure. While factors such as age, sex, initial pain severity, or the presence of neurological signs like motor weakness or hypoesthesia showed no statistically significant association with symptom improvement or recurrence, these findings are consistent with those of a previous study, which also reported no correlation between patient characteristics and symptom improvement [31]. Interestingly, however, the coexistence of both neck pain and radicular pain emerged as a highly significant predictor of symptom recurrence. This indicates that patients with combined pain presentations might require more tailored or intensive treatment strategies. Furthermore, these results indicate the broad applicability of US-guided cervical SNRB across diverse patient subgroups, potentially expanding its clinical utility. Conversely, the lack of correlation between symptom relief and MRI findings challenges the conventional reliance on imaging results for treatment decisions. This suggests that clinical outcomes may not always correspond with anatomical abnormalities observed on MRI [32].

Another point to consider is the results concerning the duration of treatment are noteworthy. Our study observed pain improvement in 61% of patients at 4 weeks, 15.9% at 8 weeks and in 15% at 12 weeks after treatment. This indicates that US-guided SNRB, which can be performed safely within a relatively short time frame, enabled some patients to avoid surgery and demonstrated efficacy even beyond 3 months. These findings suggest a potentially longer duration of effect compared to previous studies, such as the study by Yamauchi et al., which reported a treatment effect lasting up to 30 days in 20 CR patients treated with US-guided nerve block [25,26].

This study has several limitations. Firstly, its retrospective design introduces selection bias and involves a relatively small sample size, limiting the generalizability of our findings. Future prospective studies with larger cohorts are needed to validate these results and explore the role of SNRB in the broader treatment landscape for cervical radiculopathy (CR). Secondly, the injected nerve root might not have been the sole pain source, as site selection was determined by experienced physicians based on symptoms, dermatomes, and MRI findings, which have shown only 28% concordance between MRI findings at the adjacent spinal level and symptoms in previous studies [32]. Therefore, a more detailed physical examination and medical history are essential. Thirdly, the follow-up period was relatively short. Additionally, variations in the technical execution by different technicians and the subjective nature of the Visual Analog Scale (VAS) may have introduced inconsistencies. Further research with a larger cohort and a longer follow-up is required to strengthen the evidence supporting the efficacy of US-guided SNRB in delaying or avoiding surgery for CR.

Consequently, our study demonstrates that ultrasound-guided selective nerve root block (US-SNRB) offers substantial symptom improvement in patients with cervical radiculopathy, positioning it as a compelling alternative to surgery. This conclusion aligns with previous research that questions the necessity and outcomes of surgical intervention for CR. Given the inherent risks and significant resource demands of surgery, US-SNRB stands out as a valuable and effective less invasive option for managing CR, reinforcing its potential to supplant surgery in suitable cases.

## 5. Conclusions

In conclusion, our study emphasizes the therapeutic effectiveness of US-guided cervical SNRB in managing CR, offering substantial symptom relief and potentially reducing the need for surgical intervention. US-guided cervical SNRB demonstrates significant promise as a procedure for CR management, as it enabled many patients to avoid or delay surgery. These findings suggest that US-guided cervical SNRB can provide meaningful pain relief and may serve as an effective conservative treatment option. Nevertheless, further large-scale research is necessary to confirm these roles and to assess the long-term efficacy and safety of this procedure.

## Figures and Tables

**Table 1:** Distribution of Different Factors in the Study Population.

Factor	N (%)
Age	
20–29	3 (3.3)
30–39	4 (4.4)
40–49	18 (19.6)
50–59	25 (27.2)
60–69	26 (28.3)
≥70	16 (17.4)
Gender	
Female	48 (52.2)
Male	44 (47.8)
Presenting symptom	
Neck pain	11 (12.0)
Radiating pain	17 (18.5)
Neck pain and Radiating pain	64 (69.6)
Diagnosis for cause of pain	
Cervical disc herniation	39 (42.4)
Cervical disc herniation and Foraminal stenosis	44 (47.8)
Cervical disc herniation and Myelopathy	9 (9.8)
Pathology level	
Cervical disc herniation	
C3-C4 only	1 (1.1)
C4-C5 only	10 (10.9)
C5-C6 only	14(15.2)
C6-C7 only	21 (25.0)
C7-T1 only	1 (1.1)
Multiple level	43 (46.7)
Foraminal stenosis	
C3-C4 only	0 (0.0)
C4-C5 only	1 (2.3)
C5-C6 only	9 (20.5)
C6-C7 only	9 (20.5)
C7-T1 only	0 (0.0)
Multiple level	25 (56.8)
Initial pain severity	
Moderate pain (VAS: 5–6)	70 (76.1)
Severe pain (VAS: ≥7)	22 (23.9)
Improvement of symptoms	
No	23 (25.0)
Yes ^a^	69 (75.0)
Recurrence	
No	44 (47.8)
Yes	48 (52.2)

^a^ The timing of improvement was as follows: 55 patients after 4 weeks, 11 after 8 weeks, and 3 after 12 weeks. Abbreviations: N, number; %, percentage.

**Table 2:** Distribution of Factors by Improvement or Recurrence.

	Improvement of Symptoms (*n* = 69)	No Improvement of Symptoms (*n* = 23)	Chi Square *p* Value	Recurrence of Symptoms (*n* = 48)	No Recurrence of Symptoms (*n* = 44)	Chi Square *p* Value
**Presenting symptom, *n* (%)**			0.124			0.008 *
Neck pain	11 (15.9)	0 (0.0)		2 (4.2)	9 (20.5)	
Radiating pain	12 (17.4)	5 (21.7)		6 (12.5)	11 (25.0)	
Neck pain and Radiating pain	46 (66.7)	18 (78.5)		40 (83.3)	24 (54.5)	
**Diagnosis for cause of pain, *n* (%)**			0.396			0.878
Cervical disc herniation	32 (46.4)	7 (42.4)		21 (43.8)	18 (40.9)	
Cervical disc herniation and Foraminal stenosis	31 (44.9)	13 (47.8)		23 (47.9)	21 (47.7)	
Cervical disc herniation and Myelopathy	6 (8.7)	3 (9.8)		4 (8.3)	5 (11.4)	

* *p* < 0.05. Abbreviations: *n*, number; %, percentage.

**Table 3:** Logistic Regression Analysis for Possible Outcome Predictors of Improvement or Recurrence.

Factor	Improvement of Symptoms			Recurrence of Symptoms		
	OR	95% CI	*p* Value	OR	95% CI	*p* Value
Age	1.022	0.982–1.063	0.285	0.984	0.951–1.017	0.333
Gender	1.000	0.389–2.572	1.000	1.200	0.529–2.724	0.663
Initial pain severity ^a^	1.169	0.395–3.462	0.778	0.702	0.268–1.836	0.471
Motor weakness	1.144	0.277–4.731	0.853	1.707	0.464–6.286	0.421
Sensory symptom (hypoesthesia)	1.144	0.277–4.731	0.853	1.707	0.464–6.286	0.421
Spurling sign	1.561	0.569–4.285	0.387	1.520	0.652–3.543	0.332
Shoulder abduction relief sign	1.000	0.389–2.572	1.000	1.707	0.747–3.902	0.205
DTR (Biceps jerk)	0.702	0.138–3.576	0.671	0.860	0.231–3.206	0.823

^a^ Moderate pain, VAS 5–6; severe pain, VAS ≥ 7. Abbreviations: OR, odds ratio; CI, confidence interval; DTR, deep tendon reflex; %, percentage.

## Data Availability

The original contributions presented in the study are included in the article, further inquiries can be directed to the corresponding authors.
